# Survival analysis of different treatment modalities and associated outcomes for canine primary pulmonary carcinoma

**DOI:** 10.3389/fvets.2026.1683766

**Published:** 2026-03-25

**Authors:** Nicole Louie, Desiree Hung, Andrea Mosca, Angel Almendros, Antonio Giuliano

**Affiliations:** 1 Department of Veterinary Clinical Sciences, Jockey Club College of Veterinary Medicine and Life Sciences, City University of Hong Kong, Kowloon, Hong Kong SAR, China; 2 Veterinary Specialty Hospital, Hong Kong, Hong Kong SAR, China; 3 Harvest Veterinary Oncology Centre, Kowloon, Hong Kong SAR, China

**Keywords:** canine, chemotherapy, metronomic chemotherapy, primary pulmonary carcinoma, surgery, tyrosine kinase inhibitors

## Abstract

Primary pulmonary carcinoma (PPC) represents about 1% of all canine malignancies. This study retrospectively evaluated the clinical presentation and survival outcomes of 39 dogs with PPC in Hong Kong across different treatment modalities. Treatment groups were categorized as “surgery alone,” “surgery and chemotherapy,” “chemotherapy alone,” and “no treatment.” Chemotherapy protocols were further classified as maximum tolerated dose and metronomic chemotherapy. Advanced-stage diseases (stage III or above) were the most prevalent (*n* = 27, 64.1%). Survival times varied significantly across treatment groups (*p* < 0.001). Dogs treated with surgery alone had the longest median survival time (568 days), while those receiving adjuvant maximum tolerated dose chemotherapy post-surgery had a significantly shorter time to progression compared to surgery alone (143 vs. 427 days, *p* = 0.013). Among non-surgical cases, chemotherapy significantly prolonged median survival time compared to no treatment (*p* = 0.006). Tumor-related mortality was highest in untreated cases (77.8%). Chemotherapy achieved stable disease in 57.1% and partial response in 28.6% of advanced-stage patients, indicating potential benefits to delay life-threatening signs and improve quality of life. Metronomic chemotherapy protocols conferred a progression free interval 3 times longer than maximum tolerated dose chemotherapy (168 vs. 52 days; *p* = 0.028). This study is the first to report survival outcomes of untreated PPC cases, and second in veterinary literature to highlight the therapeutic potential of metronomic chemotherapy in managing advanced-stage canine PPC. Further prospective studies are warranted to evaluate the efficacy and toxicity profiles of individual therapies.

## 1 Introduction

Primary lung tumors are uncommon in dogs, accounting for approximately 1% of all canine malignancies (1–3). The majority of canine lung tumors are carcinomas, collectively referred to as primary pulmonary carcinomas (PPC) (4). Adenocarcinomas are the most common subtype, comprising approximately 75% of cases, whereas other carcinoma, and squamous cell carcinoma collectively comprise the remaining 13–15% of primary epithelial lung tumors (4–6). Accurate staging of PPC is critical for prognostication and treatment planning. A revised TNM-based staging system, canine lung carcinoma stage classification (CLCSC), specific to canine PPC was recently introduced, adapted from human lung cancer staging (7). This system integrates clinical evaluation and diagnostic imaging, such as thoracic radiography or computed tomography. It has demonstrated improved prognostic accuracy, validated in a large cohort study (7, 8).

Survival in dogs with primary pulmonary carcinoma is influenced by multiple prognostic indicators, including lymph node (LN) status, tumor stage, clinical presentation, tumor burden, age, and histopathological characteristics. LN metastasis is consistently associated with poorer outcomes, with markedly shorter median survival times (MST) compared with LN-negative disease (2, 8–11). Early-stage, solitary tumors (T1) confer substantially longer survival than multifocal disease or tumors invading adjacent structures (6, 8). The presence of clinical signs and increasing age are also associated with reduced survival (6, 12). Histopathology plays a critical prognostic role, as well-differentiated tumors and papillary adenocarcinomas, particularly at an early clinical stage (T1N0M0), are associated with significantly longer MST and disease-free intervals compared with other tumor types and higher-grade or more advanced disease (6, 8).

Surgical resection remains the primary treatment for localized PPC, displaying longest median survival times depending on tumor size, lymph node involvement, and histologic subtype (2, 6, 13–15). However, many cases are inoperable or recur after surgery (14). Maximum tolerated dose (MTD) chemotherapy is often pursued as an adjuvant or alternative treatment with inconsistent efficacy reported (15–22). Metronomic chemotherapy (MC), an approach involving continuous, low-dose oral chemotherapeutic agents, offers a promising alternative with reduced toxicity and delayed tumor progression (23). To date, only one study has evaluated MC in canine PPC, reporting prolonged survival and slowed disease progression compared to surgery, MTD chemotherapy, or palliative care (14).

PPC is poorly characterized in Asian canine populations, with only one published study from Japan (12). Large breed dogs commonly diagnosed with PPC in Western countries, such as Australian Shepherds, Boxers, and Bernese Mountain Dogs are less prevalent in Asia, where smaller breeds predominate (1, 24–27). Similarly, human non-small cell lung cancer (NSCLC) which shares clinical and biological features with canine PPC, exhibits distinct characteristics in Asian compared to Western cohorts (12, 28–30). These parallels underscore the potential for regional differences in canine PPC, highlighting the need for region-specific studies to evaluate clinical outcomes and treatment responses in canine PPC.

This study aims to provide insights into management of canine PPC in Asian veterinary populations through (1) describing the clinical presentation of canine PPC in Hong Kong, (2) comparing survival time and time to progression among treatment modalities, and (3) compare progression free intervals specific to different chemotherapy protocols. We hypothesize that dogs with surgically resected PPC will demonstrate survival outcomes comparable to previous studies in western countries and that MC prolong survival and delay tumor progression in dogs with advanced, non-surgical, recurrent, or metastatic PPC.

## 2 Materials and methods

### 2.1 Case selection criteria

A retrospective review was conducted on the medical record database of three multidisciplinary referral centers with concurrent availability of board-certified oncology and surgery service in Hong Kong. The review encompassed all canine patients diagnosed with primary pulmonary neoplasia from March 2008 to December 2024. Informed consent was obtained from all dog owners for the use of anonymized clinical information for research and publication purposes. All clinical care and treatment by licensed veterinarians are in accordance with established standards of veterinary best practice and consented by owners. Cases were included if a confirmed diagnosis of primary pulmonary carcinoma (PPC) was made by a board-certified pathologist, histologically or cytologically, and if the patient had a complete medical record with a post-diagnostic adequate follow-up period. Patients were excluded from the study if they had other advanced pulmonary or cardiac diseases that were potentially life-threatening, failed to recover from surgery, or had an insufficient post-diagnostic follow-up period (<60 days).

### 2.2 Data collection

Data extracted included patient demographics (age, sex, breed, spay/neuter status), clinical presentation, diagnostic methodology, tumor characteristics (size, clinical stage, recurrence, metastatic sites), and treatment details. Tumor size, defined as the maximum dimension of the primary pulmonary lesion, was determined from the largest diameter recorded before fixation of formalin. In cases without surgical removal, measurements from three-views thoracic radiography and abdominal ultrasound with or without computed tomography (CT) were used. Clinical staging was based on the results of baseline diagnostics performed at the time of diagnosis, which included hematology, biochemistry, urinalysis, and either TBCT or thoracic radiographs. The staging classification followed the human-adapted canine lung carcinoma stage classification (CLCSC) proposed by Lee et al. (Supplementary Table 1) (7).

Based on the treatment they received, patients were categorized into one of four groups: “surgery alone,” “surgery and chemotherapy,” “chemotherapy alone,” or “no treatment.” Treatment modalities were determined according to recommendations made by board-certified oncologists, informed by the clinical severity and progression of disease at the time of diagnosis. Non-surgical PPC comprised cases in which surgery was not performed after clinical and imaging evaluation, informed by disease extent including regional and distant metastases. Patients receiving only supportive care (e.g., palliative medications like non-steroidal anti-inflammatory drugs, corticosteroids, or antibiotics) were categorized under the “no treatment” group to allow comparison of survival outcomes across all treatment modalities.

### 2.3 Outcome parameters

For cases that underwent surgery, surgical and histopathological reports were reviewed to obtain details including the date of surgery, visible tumor adhesion or invasion into adjacent structures, histological type, completeness of excision, and mitotic count (in 10 consecutive high-power fields) as defined in Meuten et al. index per high-power field (31). Complete excision was defined as histologically complete margins as reported by the attending pathologist.

For patients who received chemotherapy, information was collected on the type and number of drugs administered, duration of treatment (in days), and whether it was given as a sole or adjuvant agent. Tumor response was assessed using the Veterinary Cooperative Oncology Group (VCOG) canine response evaluation criteria for solid tumors (cRECIST v1.0) (32). Responses were categorized as complete response (CR), partial response (PR), stable disease (SD), or progressive disease (PD) based on standardized definitions (Supplementary Table 2).

Key outcome parameters included: time to progression (TTP), median survival time (MST), progression-free interval (PFI) and causes of death. TTP was defined as the interval between the date of surgery or initiation of chemotherapy and the earliest documented event of tumor recurrence, progression, or tumor-related death. MST was defined as the median time from diagnosis to tumor-related death or last follow-up. The treatment-specific PFI was defined as the time from the first administration of chemotherapy to tumor recurrence, progression, or tumor-related death.

### 2.4 Data analysis

The clinical presentation and tumor characteristics of the dogs were descriptively analyzed. All statistical analyses were performed using SPSS Statistics version 29 (IBM, New York, United States). Categorical variables were presented as numbers and percentages, whereas continuous variables were reported as median and range. The chi-square (χ^2^) test or Fisher’s exact test was used to compare stage distribution among different treatment groups due to categorical nature of the data.

Median progression-free interval (PFI), median time to progression (TTP), and median survival time (MST) were estimated using the Kaplan–Meier estimator, with 95% confidence intervals. Documented progressive disease and death from tumor related causes were considered events for TTP and MST analysis, respectively, in the Kaplan–Meier estimator. Dogs were censored if they were still alive or lacked documented progressive disease by the end of the study period. For PFI analysis, tumor recurrence, progression, or tumor-related death was considered events, while cases were censored if chemotherapy continuation was unknown due to loss to follow-up.

## 3 Results

### 3.1 Case selection results

A total of 39 client-owned dogs with a diagnosis of primary pulmonary carcinoma (PPC) matched the inclusion criteria and were included. Patient and tumor characteristics are summarized in Table 1. Poodles (*n* = 7), Mongrels (*n* = 4), and Schnauzers (*n* = 4) were the most common breeds affected. The study population consisted of 16 spayed females and 23 males (21 neutered). The median age at diagnosis was 11 years and 10 months (range, 6 years 2 months–15 years).

**Table 1:** Demographics and tumor characteristics of 39 patients with primary pulmonary carcinoma.

Characteristics (*n* = 39)	Categories	Number (range)
**Age at diagnosis**	Median (range)	11 years 10 months (6 years 2 months–15 years)
**Sex**	Male neutered	23 (59.0%)
	Neutered	21 (91.3%)
	Intact	2 (8.7%)
	Female	16 (41.0%)
	Spayed	16 (100%)
	Intact	0 (0%)
**Breed**	Pure breed	35 (89.7%)
	Poodle	7 (20.0%)
	Schnauzer	4 (11.4%)
	Corgi	3 (8.6%)
	Pekingese	3 (8.6%)
	Shih Tzu	3 (8.6%)
	Others	15 (42.9%)
	Mix breed	4 (10.3%)
**Presenting sign(s) at diagnosis**	Symptomatic	32 (82.1%)
	Coughing	19 (59.4%)
	Dyspnoea	3 (9.4%)
	Panting	3 (9.4%)
	Lethargy	2 (6.3%)
	Others	5 (15.7%)
	Asymptomatic	7 (17.9%)
	Malignant pleural effusion	9 (23.1%)
**Primary tumor maximum diameter (cm)**	≤ 3 cm	4 (10.3%)
	3–5 cm	13 (33.3%)
	5–7 cm	10 (25.6%)
	>7 cm	7 (17.9%)
	Unknown	5 (12.8%)
**Histological classification (*n* = 24)**	Adenocarcinoma	20 (83.3.8%)
	Papillary subtype	11 (55.0%)
	Bronchioloalveolar subtype	3 (15.0%)
	Unclassified	6 (30.0%)
	Carcinoma (unclassified)	2 (8.3%)
	Pleomorphic carcinoma	1 (4.2%)
	Anaplastic squamous cell carcinoma	1 (4.2%)

Thirty-two dogs (82.1%) were symptomatic at presentation (Table 1). Coughing was the most common clinical sign (*n* = 19, 48.7%), followed by dyspnea (*n* = 3, 7.7%), panting (*n* = 3, 7.7%), and lethargy (*n* = 2, 5.1%). Malignant pleural effusions were present in 23.1% of cases (*n* = 9) at the time of diagnosis. 17.9% (*n* = 7) of the patients were asymptomatic with PPC as an incidental finding, and 71.4% (*n* = 5) of these patients were diagnosed as Stage II.

Twenty-four dogs (61.5%) were diagnosed with histopathology, while the rest (*n* = 15, 38.5%) were diagnosed cytologically. Histological and cytological assignment of tumor subtypes did not shift over the data collection period. Adenocarcinoma was the most common histological subtype (*n* = 20, 83.3%), of which 11 (55.0%) were classified as papillary and 3 (15.0%) as bronchioloalveolar. Other less common subtypes are summarized in Table 1.

Advanced stages were the most common while early stages were rare. Thirty-six (92.3%) dogs were staged using thoracic computed tomography (CT) in combination with abdominal ultrasonography and thoracic radiography, while the remaining cases were staged using thoracic radiography and abdominal ultrasonography alone. Fifteen dogs (38.5%) were assigned to stage IV with a diagnosis of distant metastases or malignant effusion (M1). Ten dogs (25.6%) were assigned to stage III with diagnosis of substage T4 without distant metastasis (M0). Twelve dogs (30.8%) were assigned to stage II, within which 41.7% were at substage T3 (*n* = 5), while only 2 dogs (5.1%) were diagnosed at stage I with ipsilateral tracheobronchial lymph node metastases (N1) detected in 3 dogs (7.7%). Fourteen out of 23 (60.9%) surgically resected PPC cases were classified as substages of T3/T4, N1, or M1. The TNM staging for all dogs grouped by treatment is summarized in Table 2A along with the significance in their distribution among treatment groups.

**Table 2A:** Tumor stages and TNM substages by the canine lung carcinoma stage classification of 39 patients with primary pulmonary carcinoma by treatment groups.

Stage/ substages	Surgery alone	Surgery + chemotherapy	Chemotherapy alone	No treatment	*p*-value
**Stage**					0.030*
I	0	2	0	0	
II	7	4	0	1	
III	4	2	2	2	
IV	2	2	5	6	
**Substage T**					0.595
T1	0	2	1	0	
T2	6	2	1	2	
T3	2	4	2	1	
T4	5	2	2	2	
Unknown^^^	0	0	1	4	
**Substage N**					0.630
N0	4	3	2	1	
N1	2	0	1	0	
Unknown^^^	7	7	4	8	
**Substage M**					0.014*
M0	11	8	2	3	
M1	2	2	5	6	

^ Patients with unknown substages were excluded from statistical analysis.

*Significant.

### 3.2 Treatment groups

The patient treatment group distribution was as follows: 13 (33.3%) surgery only, 10 (25.6%) surgery and chemotherapy, 7 (18.0%) chemotherapy only, 9 (23.1%) no treatment.

#### 3.2.1 Surgery; surgery and chemotherapy group

Of the 39 dogs, 23 (60.0%) underwent surgical resection of the tumor and the affected lung lobe. Ten dogs (43.5%) had concurrent intraoperative tracheobronchial lymph node biopsy, revealing 5 cases (21.7%) of lymph node metastasis on histology. Adhesion to adjacent structures was observed in 12 dogs (52.1%) intraoperatively, with pleural involvement (*n* = 4, 17.4%) and pericardial invasion (*n* = 4, 17.4%) being the most common. Complete surgical margins were achieved in the majority of cases (*n* = 19, 82.6%). Partial resection was reported in 2 cases (8.7%), while the status of resection margins was undocumented in 2 cases (8.7%). The median mitotic count was 14 per 10 high-power field, with a range of 1 to 50. Mitotic counts of the resected tumor of 23 surgical PPC patients distributing among treatment groups are summarized in Table 2B.

**Table 2B:** Mitotic count (in 10 high power fields) of 23 surgical patients with primary pulmonary carcinoma by treatment groups.

Mitotic count (in 10 HPFs)	Surgery alone	Surgery + chemotherapy	*p*-value
			0.894
1–10	5	4	
11–20	5	3	
>20	3	3	

^ Patients with unknown substages were excluded from statistical analysis.

*Significant.

Recurrence following surgery occurred in 14 dogs (60.9%), with the majority presenting as local recurrence within the pulmonary parenchyma on post-operative thoracic radiographs (*n* = 10, 71.4%). Notably, 85.7% (*n* = 12) of surgical patients with recurrence had PPC substages of T3/T4, N1, or M1 pre-operatively. The median time to recurrence for all surgical cases was 164.5 days (range, 17–832 days). Distant metastases to contralateral lung lobe or extrathoracic sites like the pleural cavity occurred in 2 cases (8.7%), while both local recurrence and distant metastasis occurred in another 2 cases (8.7%). Completeness of tumor excision determined in histopathology (*p* = 0.551) and mitotic count (*p* = 0.562) were not significantly associated with post-operative recurrence.

Within surgery and chemotherapy group (*n* = 10), 7 dogs (30.4%) received adjuvant chemotherapy after surgical resection of tumor without evidence of recurrence. Of these, 6 (85.7%) were treated with maximum tolerated dose (MTD) chemotherapy protocols, which included vinorelbine (*n* = 3), carboplatin (*n* = 1), a combination of carboplatin and bleomycin (*n* = 1), and doxorubicin (*n* = 1). One dog (14.3%) received metronomic chemotherapy (MC) comprising cyclophosphamide and thalidomide. Following tumor recurrence, 3 dogs (13.0%) received chemotherapy, all of which were managed with MC regimens incorporating cyclophosphamide/chlorambucil, thalidomide and non-steroidal anti-inflammatory drugs (NSAIDs) like meloxicam, with or without tyrosine kinase inhibitor (TKI) toceranib.

Only 3 of the 13 dogs from the surgery alone group (23.1%) died from tumor related causes, whereas 6 out of 10 of patients receiving both surgery and chemotherapy (60.0%) died due to PPC-related disease.

#### 3.2.2 Chemotherapy group

Seven dogs (18.0%) were managed with chemotherapy as the sole therapeutic modality. Two of these patients (28.6%) were asymptomatic while another 2 (28.6%) had pleural effusion at presentation. All received metronomic chemotherapy (MC), with combinations of cyclophosphamide (*n* = 5), chlorambucil (*n* = 1), thalidomide (*n* = 1), and lomustine (*n* = 1), 71.4% of which also received TKI (toceranib, *n* = 5) and NSAIDs (meloxicam, *n* = 5). Among those, 2 dogs (28.6%) also received maximum tolerated dose (MTD) chemotherapy, administered at time points distinct from MC treatment. The regimens included doxorubicin in 1 case and a combination of carboplatin and bleomycin in the other.

The overall tumor response was documented in 6 of the 7 patients. Four dogs (57.1%) achieved stable disease (SD) for at least 6 weeks with chemotherapy, and 2 dogs (28.6%) achieved a partial response (PR), both of which were managed with MC and TKI. Three dogs (42.9%) died of tumor-related causes.

#### 3.2.3 No treatment group

Nine dogs (23.1%) did not receive anti-cancer treatment. All these patients (100%) were symptomatic while 4 (44.4%) had pleural effusion at presentation. Seven received no intervention, while 2 were managed with palliative care with piroxicam, anti-inflammatory dose of prednisolone, and enrofloxacin. Tumor-related mortality observed in 7 dogs (77.8%). Three experienced natural death due to clinical decline, while 4 were euthanized at a terminal stage due to severe progression of clinical signs, including dyspnea, cardiopulmonary arrest, and profound lethargy.

### 3.3 Outcome comparison

The median survival time (MST) and time to progression (TTP) of patients stratified by stage, mitotic count, and treatment groups were reported along with their statistical significance (Tables 3A, 3B).

**Table 3A:** Median survival times (MST) of 39 patients with primary pulmonary carcinoma by stages and treatment groups.

Comparison groups	MST (days; 95% CI)	*p*-value
**Stage (*n* = 37)^^^**		0.003*
II (*n* = 12)	594 (205–983)	
III (*n* = 10)	361 (52–670)	
IV (*n* = 15)	62 (0–140)	
**Treatment group (*n* = 39)**		<0.001*
Surgery alone (*n* = 13)	568 (287–849)	0.554
Surgery + chemotherapy (*n* = 10)	361 (0–830)	
Chemotherapy alone (*n* = 7)	185 (135–235)	0.006*
No treatment (*n* = 9)	24 (0–77)	

CI, confidence interval. *Significant.

^ Stage I patients (*n* = 2) were excluded from statistical analysis due to a small sample size.

**Table 3B:** Median times to progression (TTP) of surgical (*n* = 22) and non-surgical (*n* = 16) patients with primary pulmonary carcinoma by treatment modalities and mitotic counts.

Comparison groups	Median TTP (days; 95% CI)	*p*-value
**Surgical patients (*n* = 23)**		0.015*
1–10 mitotic count per 10 HPFs (*n* = 9)	832 (392–1,272)	
11–20 mitotic count per 10 HPFs (*n* = 8)	151 (10–291)	
>20 mitotic count per 10 HPFs (*n* = 6)	151 (47–255)	
**Surgical patients (*n* = 22)^#^**		0.013*
Surgery without adjuvant^^^ chemotherapy (*n* = 16)	427 (169–685)	
Surgery with adjuvant^^^ MTD chemotherapy (*n* = 6)	143 (15–271)	
**Non-surgical patients (*n* = 16)^##^**		0.093
Chemotherapy alone (*n* = 7)	74 (66–82)	
No treatment (*n* = 9)	24 (0–77)	

CI, confidence interval; MTD, maximum tolerated dose; HPFs, high power fields. *Significant.

# No significant difference in stage distribution was found among surgical patients treated with or without adjuvant chemotherapy (*p* = 0.538). Surgical patient treated with adjuvant metronomic chemotherapy (*n* = 1) was excluded from statistical analysis.

^ Adjuvant chemotherapy is defined as the use of chemotherapy after surgery without evidence of tumor recurrence.

## No significant difference in stage distribution was found among non-surgical patients treated with or without chemotherapy (*p* = 0.652).

**Figure 1:**
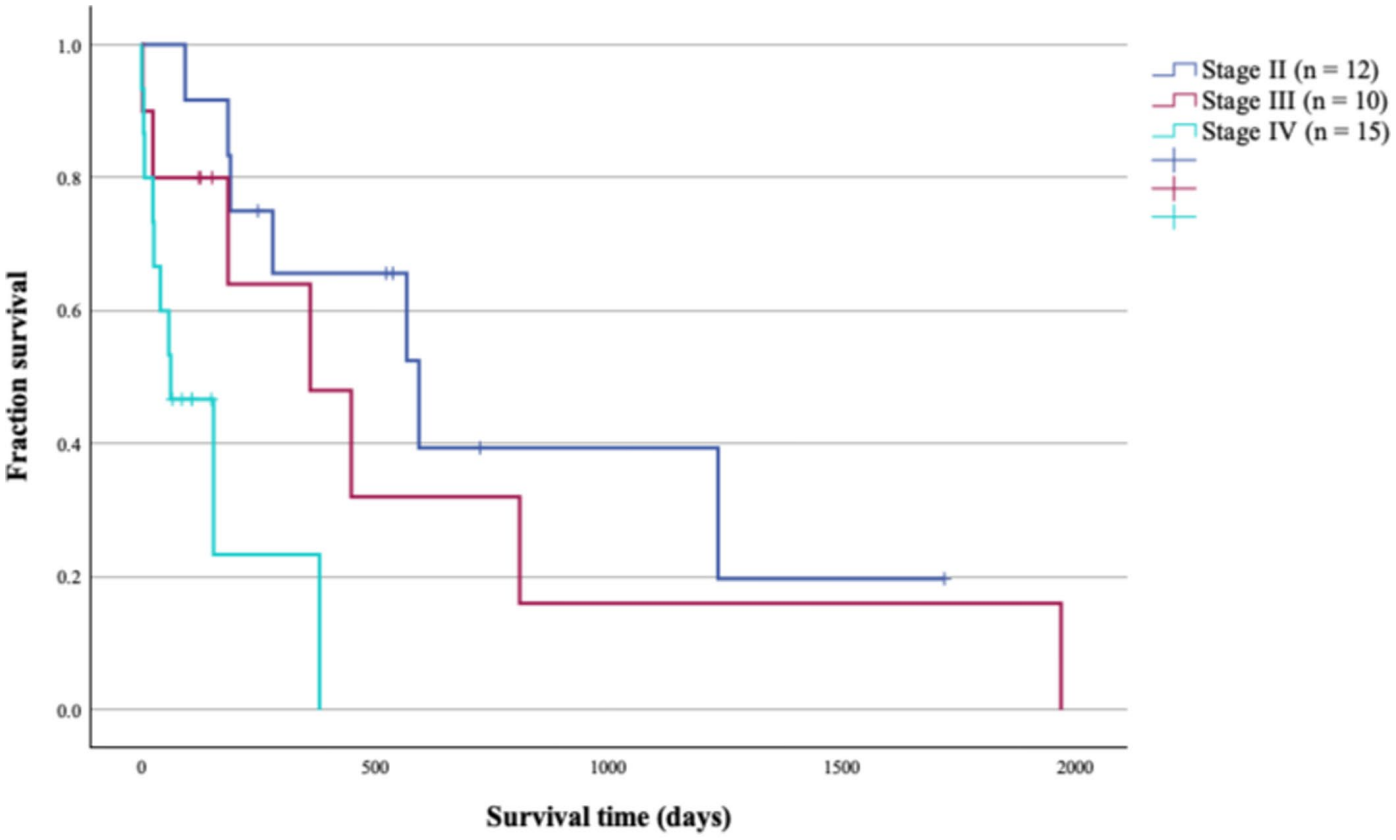
Survival times (ST) of 39 patients with primary pulmonary carcinoma stratified by stage. There was a statistical difference in median ST of patients from stage II to IV (*p* = 0.003).

Survival outcomes differed significantly across disease stages. The MSTs for dogs diagnosed with stage II (*n* = 12), stage III (*n* = 10), and stage IV (*n* = 15) PPC were 594 days, 361 days, and 62 days respectively, with a statistically significant difference observed among these groups (*p* = 0.003; Table 3A; Figure 1).

**Figure 2:**
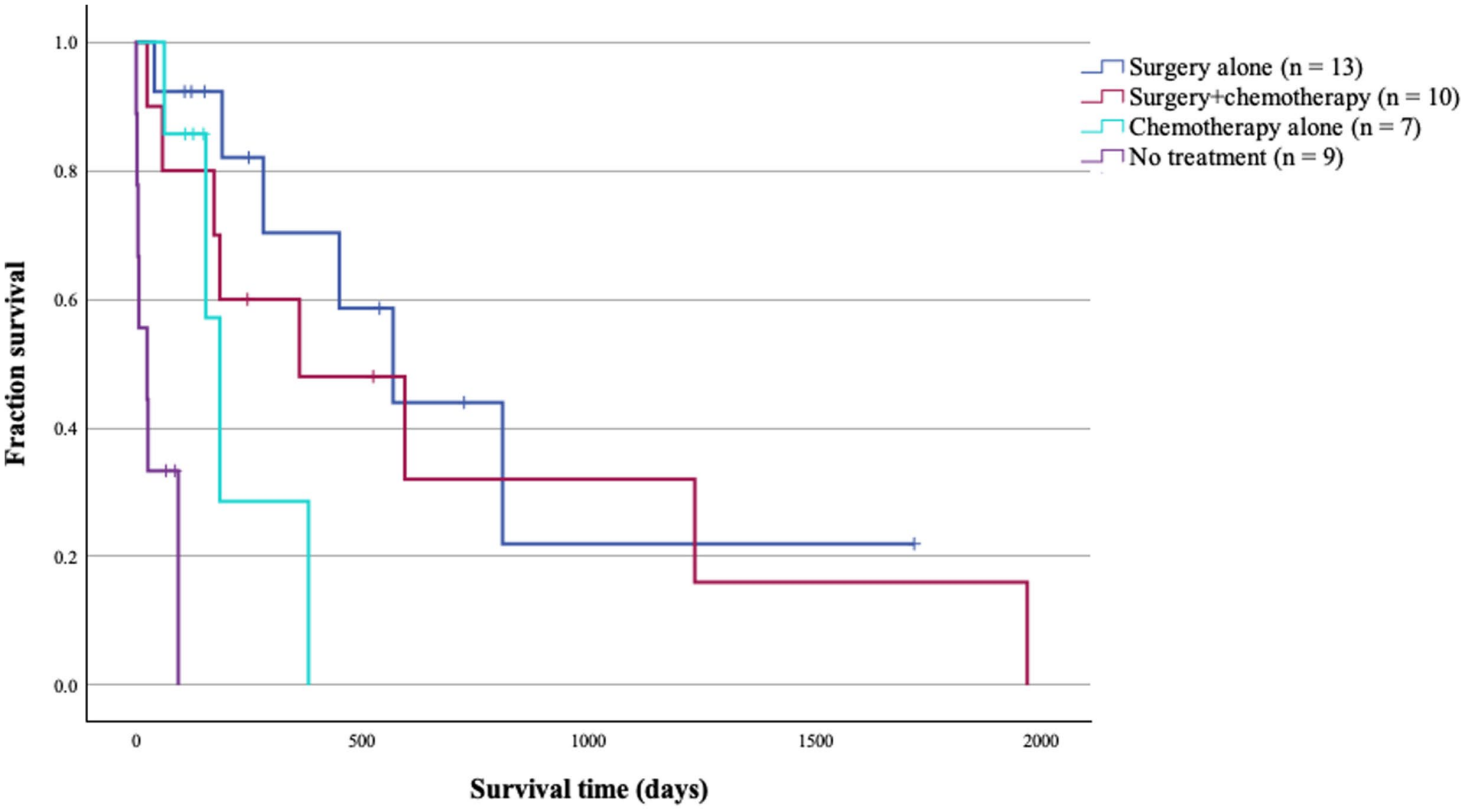
Survival times (ST) of 39 patients with primary pulmonary carcinoma stratified by treatment modalities. There was a statistical difference in median ST of patients among all 4 treatment groups (*p* <0.001).

**Figure 3:**
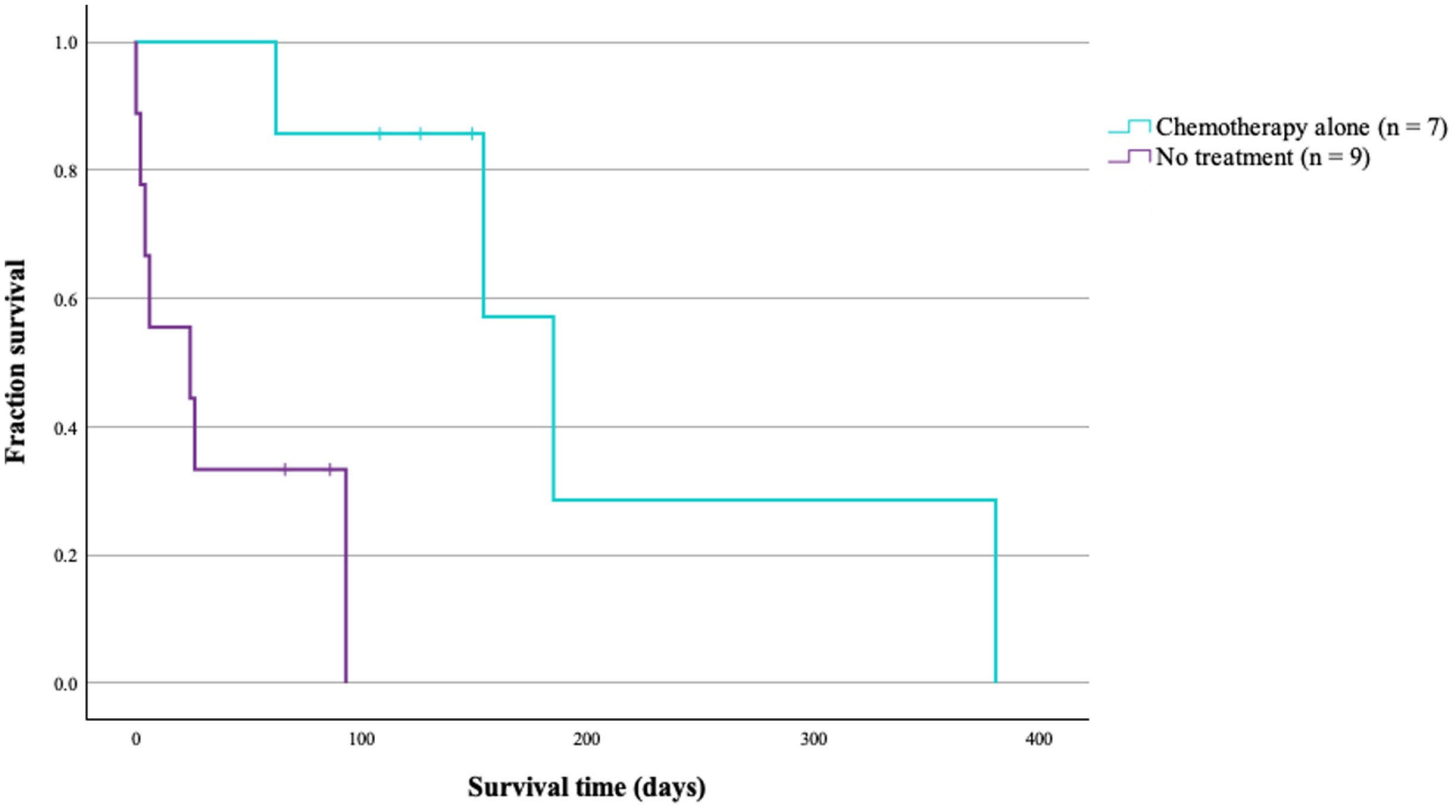
Survival times (ST) of non-surgical patients with primary pulmonary carcinoma (n = 16) treated with or without chemotherapy. The median ST of patients managed with chemotherapy was significantly longer (*p* = 0.006).

Survival outcomes also differed significantly across all 4 treatment groups (*p* < 0.001; Table 3A; Figure 2). Dogs treated with surgery alone (*n* = 13) had the longest MST at 568 days, followed by surgery and chemotherapy (*n* = 10) at 361 days, chemotherapy alone (*n* = 7) at 185 days, and no treatment (*n* = 9) at 24 days. Among surgical cases, surgery alone had a 1.5-fold longer MST than surgery and chemotherapy group; however, this difference was not statistically significant (*p* = 0.554; Table 3A). Among non-surgical cases, dogs treated with chemotherapy alone had a significant 8-fold difference to those who received no treatment (*p* = 0.006; Table 3A; Figure 3). Importantly, there was no significant difference in disease stage distribution between these 2 groups (Fisher’s exact test, 2-sided *p* = 0.542).

**Figure 4:**
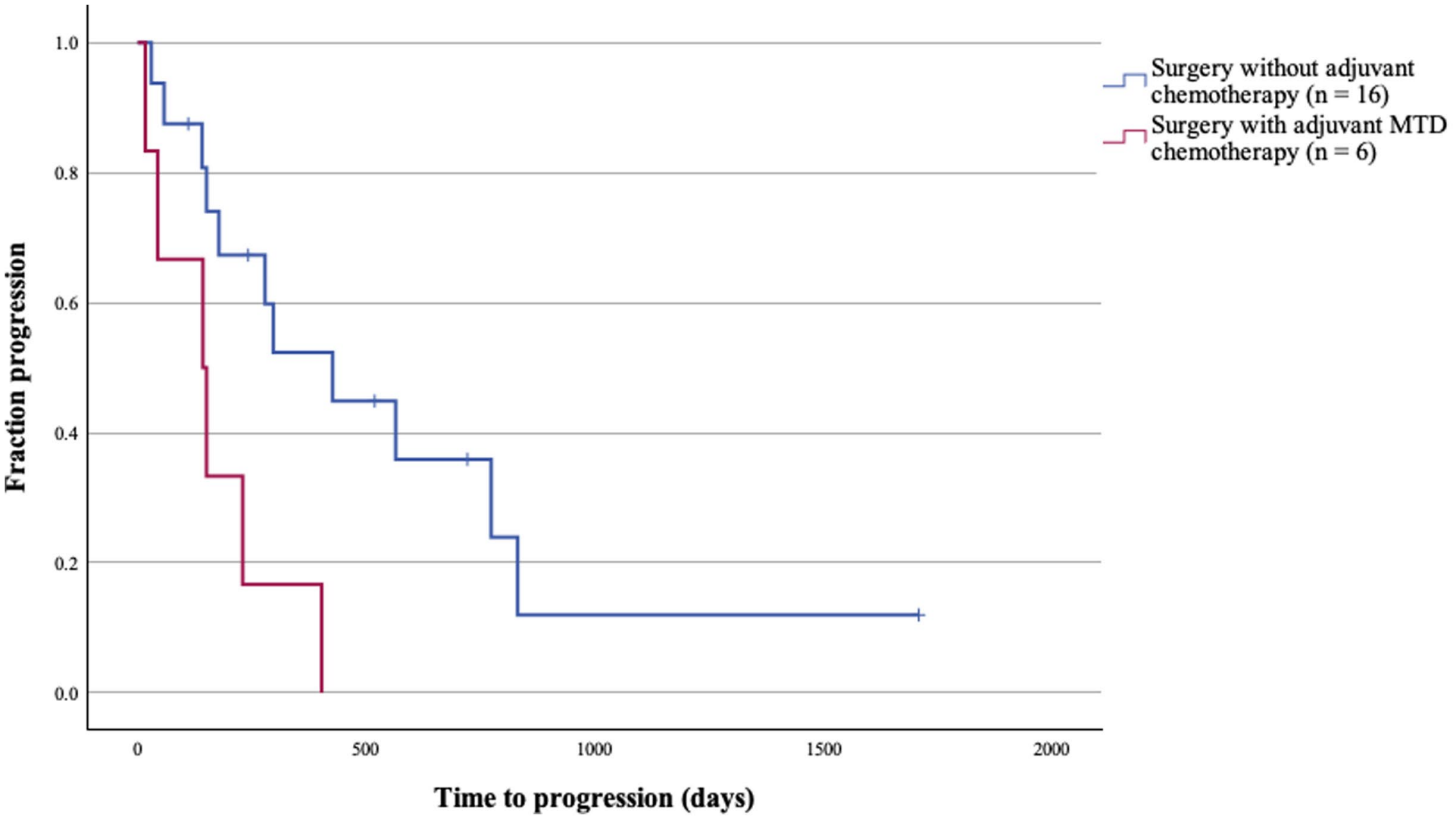
Times to progression (TTP) of surgical patients with primary pulmonary carcinoma (n = 22) treated with or without adjuvant maximum tolerated dose (MTD) chemotherapy. The median TTP of patients managed with surgery alone was significantly longer (*p* = 0.013).

The median TTP among surgical PPC cases was 427 days for surgery alone patients (*n* = 16) and 143 days for those receiving surgery with MTD adjuvant chemotherapy (*n* = 6), which is 2 times shorter compared to those managed with surgery alone (*p* = 0.013; Table 3B; Figure 4). A high mitotic count >20 per HPFs was also associated with significantly shorter TTP among resected PPC (*p* = 0.015; Table 3B). Among non-surgical PPC cases, although statistically non-significant, median TTP was 74 days for chemotherapy alone group, and 24 days for no treatment group (*p* = 0.093; Table 3B).

**Figure 5:**
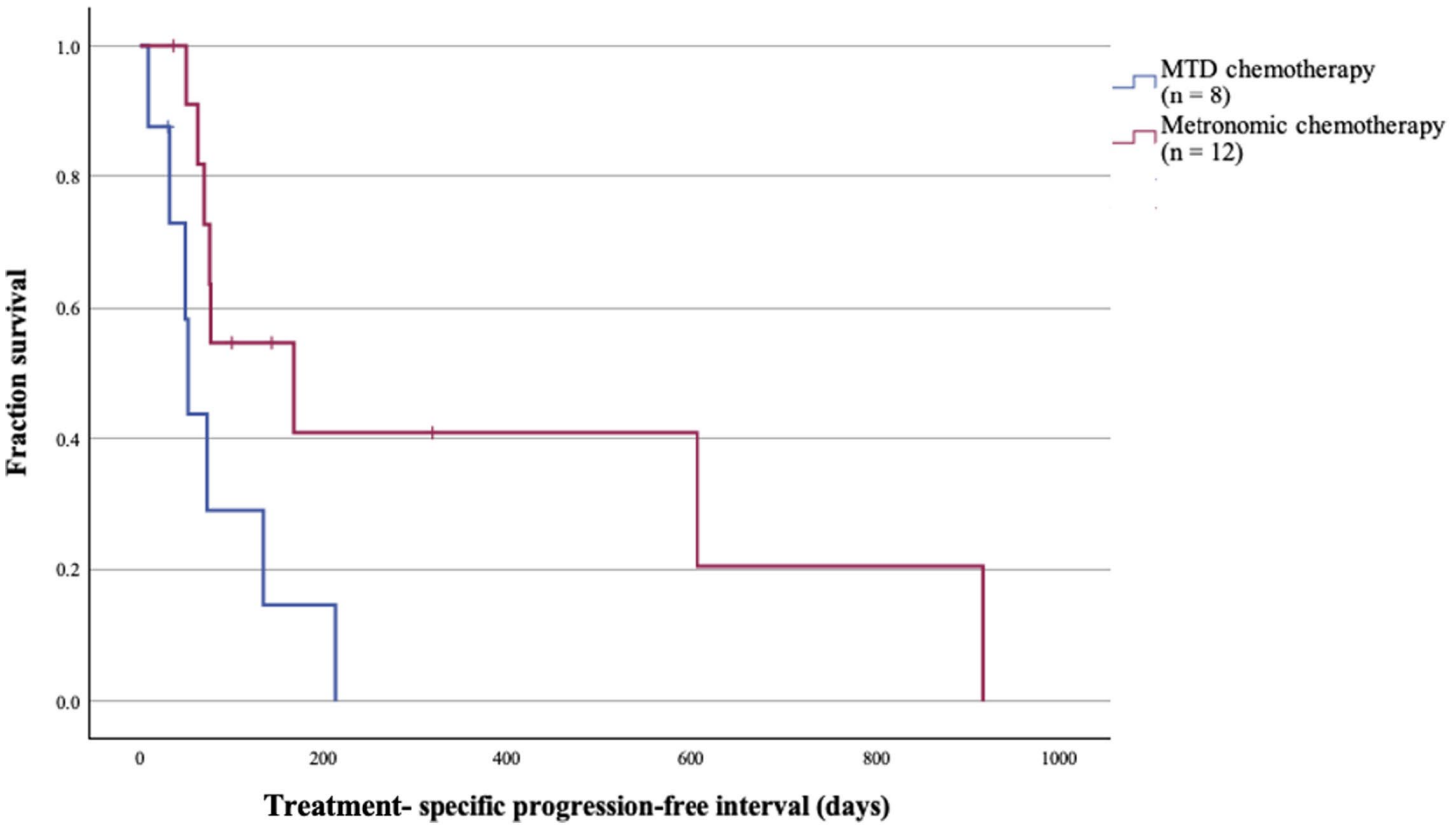
Treatment-specific progression-free intervals (PFI) of patient receiving maximum tolerated dose (MTD) or metronomic chemotherapy (MC) protocols. The median PFI of patients receiving MC regimes was significantly longer (*p* = 0.028).

Treatment-specific progression-free intervals (PFIs) also demonstrated a significant difference. Dogs treated with MTD regimes (*n* = 8) had a median PFI of 52 days, whereas those managed with MC protocols (*n* = 12) had a 3-fold longer PFI of 168 days (*p* = 0.028; Figure 5).

## 4 Discussion

This study provides critical insights into management of primary pulmonary carcinoma (PPC) in Hong Kong. Our findings align with previous studies, demonstrating a predominance of older dogs with a median age of 11 years at diagnosis (1, 24, 25, 33). While Western studies frequently report large and giant breeds overrepresented, our study population showed a predominance of small-to-medium sized dogs, with Poodles being the most frequently affected. This finding may be a reflection of the general dog population in Hong Kong rather than a true breed predisposition, as observed in other Asia-based studies (12, 34).

Approximately half of PPC patients in our study presented with coughing as the leading clinical sign, consistent with previous reports, likely attributable to the space-occupying effect of PPC on the lower respiratory tract (1, 4, 8, 9, 15, 35). Notably, 17.9% of cases were asymptomatic and diagnosed incidentally. Previous reports suggested up to one-third of PPC patients can exhibit no clinical signs, reflecting the disease’s slow progression and the compensatory reserve of functional lung parenchyma (1, 5, 8, 36).

Our findings demonstrated a significant reduction in median survival time (MST) across disease stages using the canine lung carcinoma stage classification (CLCSC) (7). Most patients in our study (38.5%) were diagnosed at stage IV, characterized by malignant effusion or distant metastasis (M1), contrasting with the stage II predominance reported in other larger PPC studies (5, 7, 11, 12). The discrepancy may be attributed to the inclusion of cytologically confirmed cases in our cohort, since advanced stage presentations are less likely to be surgical candidates and are often diagnosed cytologically (14, 37). Similar findings were reported by Polton et al., observed a high proportion of patients with substage M1 diagnoses with inclusion of cytologically confirmed cases (14). Despite the prevalence of advanced-stage presentations in clinical practice, they remain underrepresented in existing veterinary literature, highlighting the need for more research into treatment and survival outcomes for dogs with advanced PPC.

Treatment outcomes in our study aligned with prior studies, with surgery alone demonstrating the longest MST, followed by surgery with adjuvant chemotherapy, chemotherapy alone, and no treatment (5, 7, 11). The surgery alone group also showed longer time to progression (TTP) compared to surgery and chemotherapy group, though the lack of improved survival or progression outcomes with adjuvant chemotherapy may reflect treatment bias. Patients with adverse prognostic indicators (e.g., higher clinical stage, lymph node or distant metastasis, pleural effusion or clinical signs at diagnosis) may be more likely to receive adjuvant chemotherapy at the discretion of the attending clinician (5). In this study, non-surgical PPC was defined as cases in which surgery was not performed following clinical and imaging assessment, reflecting disease extent including regional or distant metastases, clinician judgment, and owner decision-making; as such, surgical resectability in this study should not be interpreted solely as a surrogate for lower clinical stage as some advanced stage also went for palliative surgical resection Therefore, a relatively poor survival outcome in these cases may reflect underlying disease severity or inherent tumor aggressiveness rather than treatment inefficacy. Conversely, dogs requiring surgery alone may represent a more favorable tumor biology, highlighting surgical resectability as a potential positive prognostic indicator in PPC.

Among surgical patients, 60.9% developed tumor recurrence, predominantly in advanced tumor substages (T3/4, N1, or M1). Aligning with previous studies, complete remission through surgery alone is uncommon in dogs with large tumors or metastatic disease, and the highest potential of curative intent appears highest in cases with small, solitary PPCs (12). While lymph node (LN) status and histological subtype are known prognostic factors, the retrospective nature of our study rendered LN status unavailable for a significant proportion of cases, and a small sample size of surgically treated dogs precluded meaningful statistical analysis of histological subtypes, limiting our ability to evaluate their impact (2, 6, 8–11).

Among non-surgical PPC patients, chemotherapy significantly extended survival with an 8-fold longer MST than no treatment cases. Importantly, the lack of significant difference in stage distribution between these groups supports the therapeutic efficacy of chemotherapy in managing non-surgical PPC. Although the difference in TTP between chemotherapy alone and untreated groups was not statistically significant, the trend aligns with prior studies (14), suggesting metronomic chemotherapy’s role in delaying tumor progression.

To our knowledge, this study is the first in Asia, and second in the veterinary literature, to report survival outcomes and causes of death in untreated PPC cases (14). It has been established that Asian societies have a reserved attitude toward euthanasia compared to Western societies, influenced by cultural, ethical, and religious perspectives, possibly contributing to a higher number of owners opting for palliative care for inoperable, invasive, or metastatic cases without oncologic treatment or electing for euthanasia (38–40).

Our study observed a high proportion (77.8%) of untreated PPC patients suffered from tumor-related deaths, either occurring naturally from tumor progression, exhibiting signs of severe dyspnea and subsequently cardiopulmonary arrest, or were euthanized at a terminal stage of disease. In contrast, tumor-related mortality was in a smaller proportion of patients in other studies. While neither surgery nor chemotherapy reliably achieved complete clinical remission, stable disease was achieved in more than half of chemotherapy patients and partial response in 2 cases. This demonstrates that metronomic chemotherapy may offer tangible clinical benefits in non-surgical PPC cases, delaying the onset of life-threatening respiratory signs, increase survival time (14).

The median survival time of advanced-stage PPC cases managed with surgery remained consistently poor (14). In such scenarios, maximum tolerated dose (MTD) chemotherapy is conventionally employed either as a sole or combined agent (15–22, 33). Vinorelbine has been proposed as a first-line treatment for stage IV canine PPC patient, owing to its ability to achieve lung concentrations up-to 300-fold higher than plasma to exert anticancer effects for human non-small-cell lung cancer (NSCLC) (20, 41). In our study, tumor recurrence occurred in 5 out of 6 dogs who received adjuvant MTD chemotherapy following surgery, further demonstrating inconsistent clinical efficacy of MTD protocols in canine PPC; nevertheless, as aforementioned, the impact of treatment bias cannot be excluded (5, 15–22, 33).

Metronomic chemotherapy (MC) has emerged as a promising alternative to conventional MTD in the management of various canine cancers (23, 42–44). In our study, the use of MC protocol achieved a progression-free interval (PFI) 3 times longer than MTD protocols, consistent with findings from Polton et al. (14). The anti-angiogenic, cytostatic and immunomodulatory properties of MC is well documented (33). MC directly target genomically stable endothelial progenitor cells, which may explain its efficacy compared to the cytotoxic effects of MTD chemotherapy, which are prone development of resistance due to tumor heterogeneity (14, 45–47). Furthermore, the relative safety of MC has been well established across a range of canine cancers, demonstrating a favorable toxicity profile (48). The ease and convenience of oral administration, together with relatively lower cost, may favor owner compliance over injectables in MTD regimes (14). These combined clinical advantages support the use of MC in maintaining comfort and preserving quality of life for dogs with advanced-stage PPC.

The incorporation of tyrosine kinase inhibitors (TKIs), such as toceranib, into MC-based protocols in our study represents a relatively novel strategy in veterinary oncology (49). In clinical practice, toceranib is valued for its broad mechanism of action, particularly its inhibition of VEGF receptors, which are overexpressed across multiple canine malignancies (49). In contrast, human oncology has long embraced more selective targeted therapies—most notably EGFR-directed TKIs for non-small cell lung cancer (NSCLC) (49).

Very little is currently known about the therapeutic relevance of EGFR inhibition in dogs. However, emerging evidence has identified overexpression of human epidermal growth factor receptor 2 (HER2) in canine PPC, raising the possibility that HER2-targeted TKIs such as lapatinib may exert meaningful anti-tumor effects in this setting (28, 50, 51). Further investigation is needed to determine whether HER2 inhibition offers clinical benefit in canine PPC.

At present, it also remains uncertain whether the clinical activity observed with MC combined with toceranib reflects additive or synergistic interactions. More rigorous, mechanistic, and prospective studies are required to clarify the individual and combined contributions of each agent to overall treatment efficacy.

Information like histological grading and resection margin was not available for all cases, and where reported, grading criteria were not applied uniformly across different pathologists. As tumor grade has been associated with prognosis in canine pulmonary carcinoma, the lack of complete and standardized grading limited its inclusion in outcome analyses and may have contributed to confounding. Tumor staging in this cohort was performed using either thoracic CT or thoracic radiography, which may have introduced staging heterogeneity between cases and treatment groups. Thoracic CT is recognized to have superior sensitivity for assessing tumor size (T stage) and detecting lymph node involvement (N stage). This limitation is especially relevant if CT was more frequently utilized in specific treatment cohorts, such as surgically managed cases. However, this limitation is minimized by the fact that the majority of cases in the present study underwent CT-based staging.

This study is subject to several important limitations inherent to its retrospective design. Foremost, substantial selection bias is unavoidable: clinicians may have preferentially chosen adjuvant chemotherapy for dogs with multiple negative prognostic factors, while others may have opted for surgery alone even in the presence of evident lymphadenopathy. Such non-random allocation of treatment introduces systematic differences between groups that cannot be fully adjusted for.

Treatment heterogeneity further complicates interpretation. A wide range of chemotherapy protocols including non-standardized regimens, tyrosine kinase inhibitors, and metronomic approaches were used at clinician discretion. These variations reflect real-world practice but limit the ability to attribute outcomes to any specific modality. Differences in clinician philosophy (e.g., preference for aggressive multimodal therapy versus conservative surgical management) likely contributed additional unmeasured confounding.

The small sample size within each treatment subgroup markedly reduces statistical power, increasing the risk of both type I and type II error and limiting the robustness of between-group comparisons. Although loss to follow-up was handled using Kaplan–Meier methods to account for censoring, attrition may still have introduced bias in survival estimates. Furthermore, the limited number of cases and outcome events precluded reliable multivariable Cox proportional hazards modeling and adjusted survival analyses, as such approaches require adequate sample size to avoid model overfitting and unstable estimates. Larger, multi-institutional studies with greater statistical power and more balanced subgroup representation would be necessary to support robust multivariable survival modeling, including Cox regression analyses, and to better control for potential confounding variables.

Furthermore, stage distribution was imbalanced across treatment groups, with advanced-stage disease disproportionately represented in non-surgical cohorts, as shown in Table 2A. This imbalance restricts the generalizability of cross-group comparisons and complicates interpretation of apparent survival differences.

Despite these constraints, inclusion of multiple referral centers and diverse clinical approaches provides a realistic snapshot of contemporary practice patterns. Nevertheless, prospective, standardized, and adequately powered studies are needed to validate these findings and clarify the true impact of specific treatment strategies.

This study offers crucial insights into the management of canine primary pulmonary carcinoma (PPC) within Asian veterinary populations. Advanced-stage diseases may be overlooked by previous studies, and in such cases, treatment by either surgery, chemotherapy, or a combination of both may effectively slow animal’s disease progression. The use of combined metronomic chemotherapy protocols with TKI shows promising clinical efficacy; however, further research is necessary to evaluate the effects and toxicity profile of individual agents.

## Data Availability

The data analyzed in this study is subject to the following licenses/restrictions: patient and client confidentiality. Requests to access these datasets should be directed to Nicole Louie, wingmlui4-c@my.cityu.edu.hk.
