# Supplementary material for: Survival analysis of different treatment modalities and associated outcomes for canine primary pulmonary carcinoma
Source: Front Vet Sci. 2026 Mar 25;13:1683766. doi: 10.3389/fvets.2026.1683766 (PMC13059658; doi:10.3389/fvets.2026.1683766)
Supplement: Supplementary file 1 [file Table_1.docx]

**Supplementary Table 1.** Human-adapted canine lung carcinoma staging classification (CLCSC) by Lee et al. (2020) (7).

| Primary  tumor (T) | Substages | Size | Distribution | Organ invasion |
| --- | --- | --- | --- | --- |
|  | T1 | ≤3cm | Solitary | None |
|  | T2 | 3–5cm | Solitary | Visceral pleura, main bronchi (not carina) |
|  | T3 | 5–7cm | Separated nodule(s) in the same lung lobe | Chest wall, pericardium,  phrenic nerve |
|  | T4 | >7cm | Separated nodule(s) in ipsilateral lung lobe(s) | Mediastinum |
| Regional lymph nodes (N) | N0 | No lymph node involvements | | |
|  | N1 | Ipsilateral tracheobronchial lymph node involvements | | |
|  | N2 | Distant lymph node involvements | | |
| Distant metastasis (M) | M0 | No distant metastasis | | |
|  | M1 | Malignant effusions, contralateral lung lobe metastases, or any extra-thoracic metastasis | | |

| Stage I | T1, N0, M0 |
| --- | --- |
| Stage II | T2–3, N0, M0 or T1–2, N1, M0 |
| Stage III | T4, N0, M0 or T3–4, N1, M0 or T1–4, N2, M0 |
| Stage IV | T1–4, N1–2, M1 |

**Supplementary Table 2.** Categories of tumor responses in the canine response evaluation criteria for solid tumors (cRECIST v1.0) established by the Veterinary Cooperative Oncology Group (VCOG) (32).

| Tumor response category | Tumor measurement |
| --- | --- |
| Complete response (CR) | The absence of the primary pulmonary tumor monitored for treatment response |
| Partial response (PR) | ≥30% reduction of the maximum tumor diameter of the primary pulmonary lesion monitored for treatment response. |
| Stable disease (SD) | <30% reduction or <20% increase of the maximum tumor diameter of the primary pulmonary lesion monitored for treatment response. |
| Progressive disease (PD) | ≥20% increase of the maximum tumor diameter of the primary pulmonary lesion monitored for treatment response. |
